# Intraoperative Measurement of Tibial Rotation With Lateral Axis Views Using C-arm for Tibial Fractures

**DOI:** 10.7759/cureus.47091

**Published:** 2023-10-16

**Authors:** Yo Kinami, Norio Yamamoto, Kazuo Fujiwara

**Affiliations:** 1 Department of Orthopedic Surgery, Okayama City Hospital, Okayama, JPN; 2 Department of Epidemiology, Graduate School of Medicine, Dentistry and Pharmaceutical Sciences, Okayama University, Okayama, JPN

**Keywords:** comminuted fracture, segmental fracture, incidence of malrotation, bimalleolar axis, tibial posterior condylar axis, tibial external rotation, tibial malrotation, c-arm

## Abstract

Malrotation of tibial fractures after intramedullary nailing remains an unsolved problem. The incidence of malrotation >10° on computer tomography (CT) measurements has been high in cases of tibial shaft fractures. We aimed to assess the accuracy of a novel method for the measurement of tibial rotation using lateral axis views of the C-arm, to prevent postoperative malrotation. Consecutive patients with fresh tibial fractures treated by intramedullary nailing between January 2021 and December 2022 were included prospectively. Baseline tibial external rotation (TER) was measured preoperatively on the non-injured normal side with CT. After proximal or distal screw fixation, the C-arm TER was measured based on lateral axis views (tibial posterior condylar axis and bimalleolar axis views). The C-arm TER was compared with the normal-side CT TER; when the difference was ≤5°, the procedure progressed, and screw fixation was carried out. The fractured-side CT TER was measured one week post-operatively. Twenty patients (13 males and seven females) were included. The mean age was 52.4 years. The Orthopaedic Trauma Association (OTA) classification was 42A in five patients, 42B in twelve patients, and 42C in three patients. The mean difference between C-arm TER and fractured-side CT TER was 2.3°±1.7°, with Pearson correlation coefficient r=0.968. The mean difference between normal-side CT TER and fractured-side CT TER was 4.8°±2.8°, and there was no incidence of malrotation >10°. The C-arm method was highly accurate in estimating CT measurements and preventing tibial malrotation.

## Introduction

Malrotation of tibial fractures after intramedullary nailing remains an unsolved problem. The acceptable range of bilateral difference in tibial rotation is ≤10° [1-5]. After tibial shaft fractures, however, rates of malrotation >10° on computer tomography (CT) measurements range from 19% to 41% [1-5]. Tibial malrotation has a potential negative impact on function, abnormal gait or osteoarthritis, and also carries risks in the realm of litigation and compensation [5-9]. Revision derotation osteotomy after nailing requires skilled surgeons and introduces additional invasiveness for patients [1,10].

Unfortunately, there has been no reliable simple method for evaluating tibial rotation during intramedullary nailing. Reports on the use of C-arm fluoroscopy to measure tibial rotation on the lateral knee and anteroposterior ankle views are limited, and a review of current literature has not confirmed its feasibility and reproducibility [11,12]. The use of cortical signs at the fracture site has been effective for simple fractures; however, it may be less useful for segmental or comminuted fractures [13]. While the use of an external tibial rotation apparatus for arthroplasty is reliable, its applicability is limited due to the requirement of the apparatus [14]. Comparing true knee and lateral ankle views between normal and fractured tibia could help prevent malrotation; however, knee joint laxity could interfere with these measurements [15]. Therefore, all previous studies have shown at least one limitation, such as lack of reproducibility, less usefulness for segmental or comminuted fractures, the necessity of additional devices, and evaluation influenced by joint laxity.

We applied bony landmarks to compare tibial rotation measurements using CT with the C-arm lateral axis views. The lateral axis views showed the same axes as the CT measurements of tibial rotation. We then developed a novel simple method using lateral axis views captured with the C-arm to carry out intraoperative measurements of tibial rotation. In the present study, we aimed to assess the accuracy of this C-arm measurement method and to evaluate whether it can contribute to the prevention of tibial malrotation.

## Technical report

Patients and study design

This prospective study for reliability analysis was conducted at a single level two trauma center. Approval from the institutional review board was obtained prior to the study. Informed consent was obtained in the form of an opt-out on the website. From January 2021 to December 2022, consecutive patients with fresh tibial fractures treated by intramedullary nailing were included in this study. The Phoenix tibial nail (Zimmer Biomet, Warsaw, IN, USA) or the TN-Advanced nail (DePuy Synthes, West Chester, PA, USA) was used. A senior orthopedic surgeon evaluated tibial rotation during and after surgery. Exclusion criteria were bilateral fractures of the tibia and previous surgery or trauma to either side, including endoprosthesis of the knee or ankle joint, osteotomies, fractures, and syndesmotic injuries.

Preoperative setting

**Figure 1:**
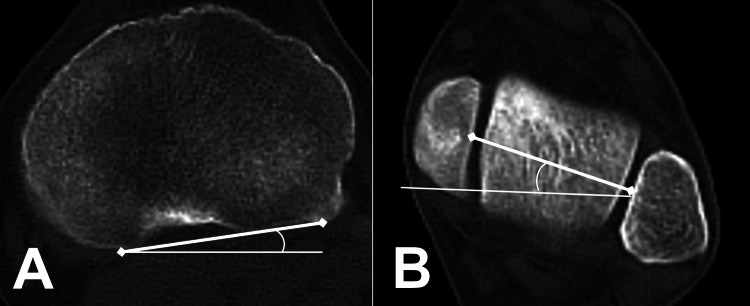
CT measurement of the angle The “tibial posterior condylar axis” was the line connecting the posterior aspects of the lateral and medial condyles at the level just above the fibular head (A). The “bimalleolar axis” was the line connecting the centers of the articular surface between the medial and lateral malleoli at a level just below the tibial plafond (B). “CT tibial external rotation” was the angle measured between the lines of the tibial posterior condylar axis and the bimalleolar axis (angle of (B) - angle of (A)). External rotation was a positive value, and internal rotation was a negative value.

In our hospital, routine CT scans are performed for all tibia fractures in the emergency room to evaluate the details and prevent missing any fracture lines. Simultaneous CT imaging of the normal tibia with the fractured tibia was performed for surgical planning. The SOMATOM Force (SIEMENS, Munich, Germany) scanner was used for CT imaging, and the Synapse Radiology Information System (FUJIFILM, Tokyo, Japan) was used to calculate the digital measurements. Tibial external rotation (TER) was given a positive value, whereas internal rotation was represented by a negative value. The normal-side CT TER was measured as the angle formed between the lines of the tibial posterior condylar and bimalleolar axes (Figure 1). The tibial posterior condylar axis and the transtibial axis had no significant difference in torsion within 2 cm of the proximal tibial articular surface [16]. The bimalleolar axis exhibited higher interobserver and intraobserver reliability than other methods [17].

**Figure 2:**
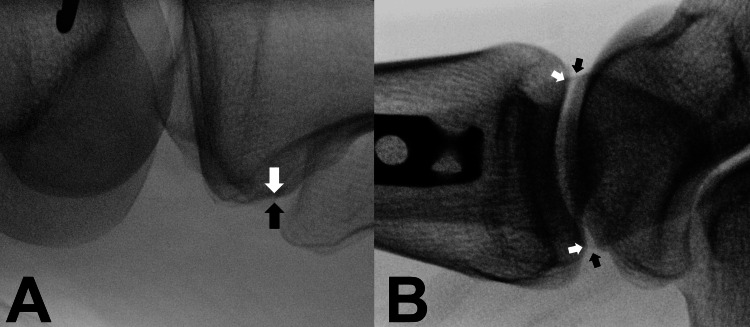
Lateral axis views Tibial posterior condylar axis view: the posterior aspects of the lateral condyle (black arrow) and the medial condyle (white arrow) were overlapped at the knee level (A). Bimalleolar axis view: the lateral malleolus was centered on the medial malleolus at the ankle joint level; the position of malleoli was recognized with the lateral malleolus cortex edge (black arrow) and plafond articular edge (white arrow) (B).

We defined the lateral axis views on the fluoroscopy image, as follows: tibial posterior condylar axis view and bimalleolar axis view (Figure 2). The tibial posterior condylar axis view was defined by the posterior aspects of the medial and lateral condyles overlapping at the knee joint level. The bimalleolar axis view was defined as the alignment where the lateral malleolus was centered on the medial malleolus at the ankle joint level. The position of the malleoli was recognized with lateral malleolus cortex and plafond articular edge.

C-arm method for measuring tibia rotation and comparing bilateral tibia rotation

**Figure 3:**
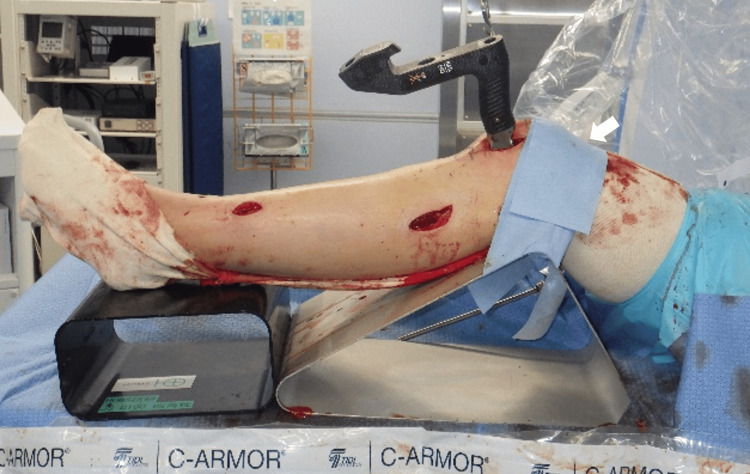
Position of the leg The fractured limb was positioned parallel to the table on radiolucent blocks for maintaining a knee flexion of 20-30°. The lateral parapatellar approach was used for nail insertion in all patients. Rotation was maintained during angle measurement by fixing the knee with surgical tape (white arrow) to the radiolucent block.

A mobile C-arm fluoroscope image intensifier with a protractor (Cios Select; SIEMENS, Munich, Germany) was used for the measurements. The patient lay in the supine position on a radiolucent surgical table (ALPHAMAXX; MAQUET, Rastatt, Baden-Wurttemberg, Germany). A lateral parapatellar approach was used to insert the nails in all patients. The fractured limb was positioned parallel to the table on radiolucent blocks to maintain a semi-extended position (knee flexion 20-30°) (Figure 3).

**Figure 4:**
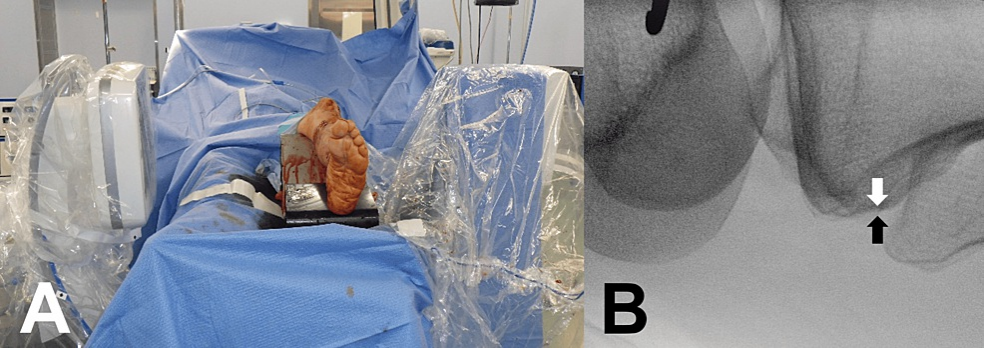
Knee lateral view (tibial posterior condylar axis view) While the C-arm was set to capture a horizontal lateral knee view (A), the fractured tibia was rotated on the longitudinal axis so that the posterior aspects of the condyles overlapped (white arrow: medial condyle; black arrow: lateral condyle) and were centered on the monitor (B).

**Figure 5:**
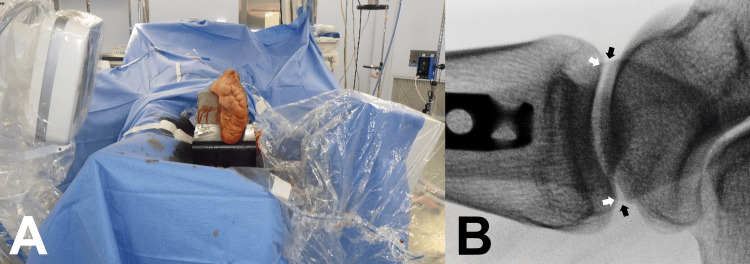
Ankle lateral view (bimalleolar axis view) The C-arm was moved parallel to the ankle joint level (A). The C-arm was rotated in the transverse plane until the lateral malleolus was centered on the medial malleolus at the level of the ankle joint and on the center of the monitor (white arrow: plafond articular edge; black arrow: lateral malleolus cortex edge) (B).

After proximal or distal screw fixation and temporary fixation of the fracture site, the C-arm was set to capture a horizontal lateral knee view. The fractured tibia was rotated on the longitudinal axis so that the posterior aspects of the condyles overlapped and were centered on the monitor (tibial posterior condylar axis view) (Figure 4). While maintaining this knee position, the C-arm was then moved to the level of the ankle joint. The C-arm was rotated in the transverse plane until obtaining the view where the lateral malleolus was centered on the medial malleolus (bimalleolar axis view); the fluoroscopic beam could then be parallel to the bimalleolar axis (Figure 5). The value of the C-arm rotation angle was defined as the C-arm TER.

**Figure 6:**
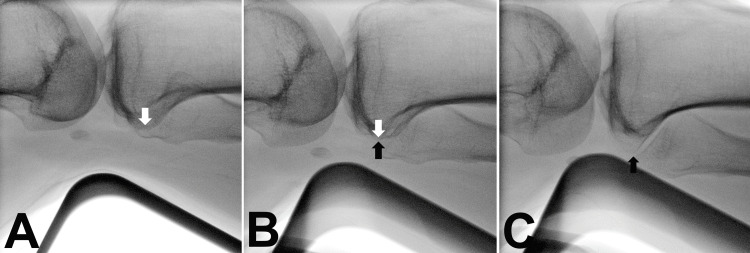
Comparing fluoroscopic image of 5° difference of knee Internal rotation 5° from tibial posterior condylar axis view (A). Tibial posterior condylar axis view (B). External rotation 5° from tibial posterior condylar axis view (C). (white arrow: medial condyle; black arrow: lateral condyle).

**Figure 7:**
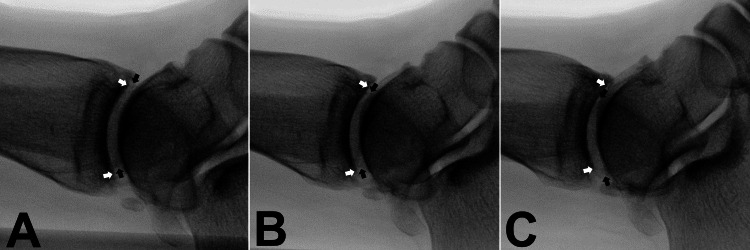
Comparing fluoroscopic image of 5° difference of ankle Internal rotation 5° from bimalleolar axis view (A). Bimalleolar axis view (B). External rotation 5° from bimalleolar axis view (C). (white arrow: medial malleolus articular edge; black arrow: lateral malleolus cortex edge)

The centered position of the bony structures on the monitor is important because of the fluoroscopic beam diffusibility. The difference between lateral axis views and 5° difference views could be recognized on fluoroscopy (Figure 6 and Figure 7).

**Figure 8:**
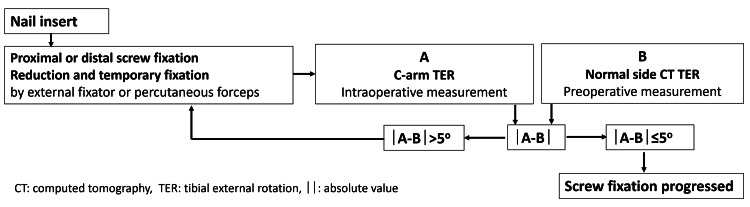
Flowchart of angle comparison and fixation

After the first measurement procedure, the C-arm TER was compared with the normal-side CT TER measured before surgery. If the difference between the C-arm TER angle and the normal-side CT TER angle was ≤5°, the procedure progressed, and screw fixation was carried out. If the difference between the C-arm TER angle and the normal-side CT TER was >5°, the fracture site was re-reduced (Figure 8). A final C-arm TER angle was measured once fixation was completed and recorded in the operative notes.

After surgery, range of motion exercises were initiated at one week while maintaining non-weight-bearing status. Postoperative CT images were obtained from all patients at one week. Partial weight-bearing began at four weeks and full weight-bearing at eight weeks for all patients.

Outcome

The primary outcome was the accuracy of the C-arm TER angle during surgery. This was assessed by comparing the intraoperative C-arm TER with the fracture-side CT TER performed one week after surgery. The secondary outcome was the incidence of malrotation. To evaluate this, the difference between the fractured-side and normal-side CT TER values was calculated one week after surgery. The limit of acceptance was set as ±5° between the C-arm TER and fractured-side CT TER on the Bland-Altman plot. This number was selected based on the individual bilateral differences in the tibial rotation of healthy subjects [18].

Statistics analysis

Descriptive statistics were reported using the mean and standard deviation of the measurements and the difference in measurements. Pearson's correlation coefficient was used to assess C-arm method accuracy. A Bland-Altman plot was used to evaluate the measure of agreement. Statistical analyses were performed using EZR analysis software v1.5 (The R Foundation for Statistical Computing) and Modified R Commander v4.0.2 (for Windows; https://personal.hs.hirosaki-u.ac.jp/pteiki/research/stat/R/).

Results

**Figure 9:**
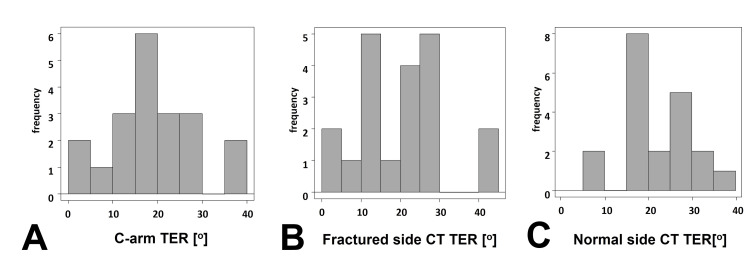
Histogram of angle measurements The C-arm tibial external rotation (TER) (A), fractured-side computed tomography (CT) TER (B), and normal-side CT TER (C).

Overall, 20 patients (13 males and seven females) were included in the study. None of the consecutive patients met the exclusion criteria. The mean age was 52.4 years (range: 20-85 years). The Orthopaedic Trauma Association (OTA) classification was 42A in five patients, 42B in twelve patients, and 42C in three patients. The mean C-arm TER was 20.7°±10.2°. The mean fractured-side CT TER was 20.7°±11.5°. The mean normal-side CT TER was 22.2°±8.1° (Figure 9).

**Figure 10:**
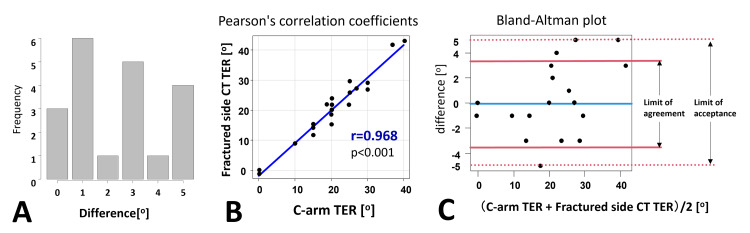
C-arm method versus CT measurement comparison The difference between C-arm tibial external rotation (TER) and fractured-side computed tomography (CT) TER [A], Pearson’s correlation coefficients [B], Bland–Altman plot [C].

The mean difference between C-arm TER and fractured-side CT TER was 2.3°±1.7°. Ten (50%) patients had differences of ≤2°. The maximum range of differences was 5°. The Pearson correlation coefficient between the two angles was r=0.968 (p<0.001), indicating a strong correlation. The Bland-Altman plot revealed that all patients had measurements within the ±5° limit of acceptance (Figure 10).

**Figure 11:**
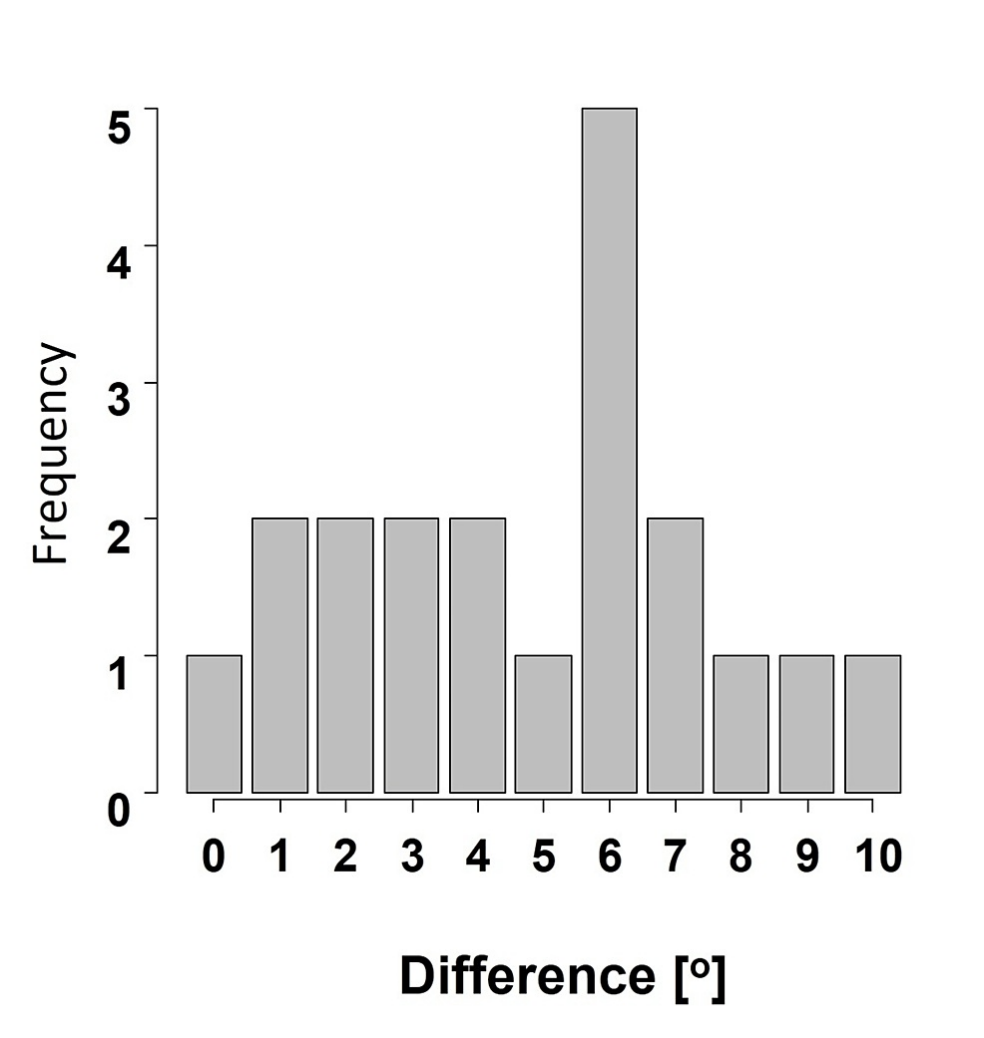
Bilateral difference of rotation Bilateral difference in tibial rotation between normal-side computed tomography (CT) tibial external rotation (TER) and fractured-side CT TER.

The mean difference between the normal-side CT TER and fractured-side CT TER was 4.8°±2.8°, and 15 (75%) patients had differences of ≤6°. The maximum difference was 10° (Figure 11). Among all, four (20%) patients required re-reduction of the fracture because the difference (specifically, 10° to 20°) between the C-arm TER angle and normal-side CT TER was >5°. There was no incidence of tibial malrotation >10°.

## Discussion

A strong Pearson's correlation coefficient between the C-arm TER and fracture-side CT TER suggested that C-arm TER is effective for intraoperative measurements of fracture-side CT TER. The mean difference between the two angle measurements was 2.3° (±1.7°). The Bland-Altman plot indicated that all patients had measurements within the ±5° limit of acceptance. We observed no cases of tibial malrotation >10°. These results suggest that the C-arm method has potential as an assessment method to prevent tibial malrotation >10°. 

To the best of our knowledge, this study is the first to assess the accuracy of angle measurements made with the C-arm. This method is useful for segmental and comminuted fractures, and it can measure the angle using a protractor-attached C-arm without other specialized devices. The tibial posterior condylar axis view is easily obtained by first recognizing the posterior aspect of the lateral tibial condyle with the fibular head (Figure 6). The bimalleolar axis can be easily recognized by the cortical line of the lateral malleolar and plafond articular edge (Figure 7).

The accuracy of this C-arm method was sufficient to estimate CT measurements and it could be attributed to the lack of interference by joint laxity in the measurements and recognition of simple bony structures for the axes. Previous studies using knee views had interference of the joint during the intraoperative evaluation compared to the CT measurement (e.g., the posterior femoral condylar view and the posterior tibial condylar line) [11,15]. Therefore, we recommend the C-arm method using the tibial posterior condylar views to evaluate tibial rotation.

This C-arm method prevented tibial malrotation >10°. Previous studies have reported a high incidence of tibial malrotation >10°, although they did not detail the methods used to prevent malrotation [1-5]. The lack of detail suggests that conventional methods were implemented. One conventional method, using anterior-posterior (AP) knee and ankle mortise views, is often performed to prevent tibial malrotation by comparing to the contralateral side [19]. This technique encompasses an increased risk for tibial malrotation in transverse, segmental, and comminuted fracture patterns, and for injuries with an ipsilateral fibular fracture [19]. Another method, assessing the cortical step sign and diameter difference sign, is also used frequently and is effective for simple fractures [13,20]. However, it is less useful for segmental or comminuted fractures. The high incidence of malrotation reported in previous studies could potentially be attributed to these two conventional methods. Inci et al. measured tibial rotation with an external tibial rotation device usually used during arthroplasty procedures [14]. They reported a mean difference of rotation between fractured and normal tibia as 4.4°(±2.6°) for 21 patients and a 0% incidence of malrotation >10°. The accuracy and results reported by them are comparable to those of the present study. However, this method requires a special device for knee arthroplasty and skill for handling. Eckart et al. described a method using lateral knee and ankle view [15]. They reported a 0% incidence of malrotation >10° in 10 patients of OTA classification 42C. Their results are comparable to that of our study, but the method involves time-consuming fluoroscopic imaging of the normal side and probable interference of knee laxity in the measurements. 

Limitations

First, normal-side CT measurements may not be an accurate reference because of individual differences in tibial rotation. The mean individual rotational difference between bilateral tibias of healthy subjects has been reported as 5.3°±4.0° [18]. Therefore, we set the limit of acceptance for our Bland-Altman plot as ±5° between the C-arm TER and fractured-side CT TER. Second, only external rotation can be measured; internal rotation cannot be measured while the C-arm rotates below the surgical table. However, internal rotation of the tibia is very rare [18]. Third, preoperative CT of the normal tibia is required. Simultaneous CT imaging of the normal tibia with fractured tibia does not incur additional time and cost in our insurance. Fourth, this method is less suitable when using the infrapatellar approach because changing the knee position would be necessary. Fifth, plateau fractures and syndesmotic injuries affect the measurements because they could potentially change the measured axes. However, no such cases were observed in this study. Sixth, the surgeon and evaluator were the same senior physicians, and we did not evaluate intra- and inter-rater reliability. There was also no third-party evaluation involving multiple centers. Ultimately, well-designed studies involving more patients, raters, fracture types, and operative approaches are necessary to clarify the reliability of the C-arm method.

## Conclusions

A simple and reliable method for evaluating tibial rotation during intramedullary nailing has not yet been established. Consequently, malrotation of tibial fractures after intramedullary nailing remains unsolved. We applied the same bony landmark axes to compare TER measurements using CT with the C-arm lateral axis views. The intraoperative method of measuring TER using the C-arm lateral axis views showed good accuracy compared to TER measured on CT. This C-arm method may be useful in efforts to prevent tibial malrotation >10°.
